# The Disease Self‐Management Among Patients With Chronic Heart Failure in an Affluent Economy: A Grounded Theory Study

**DOI:** 10.1002/nop2.70302

**Published:** 2025-10-08

**Authors:** Kakit Lam, Aimei Mao

**Affiliations:** ^1^ Kiang Wu Hospital Macau Macau; ^2^ Kiang Wu Nursing College of Macau Macau Macau

**Keywords:** chronic heart failure, grounded theory, Macau, self‐management

## Abstract

**Aims:**

To examine the difficulties patients with chronic heart failure face in managing their condition, as well as the coping strategies they employ to overcome these difficulties.

**Design:**

Grounded theory qualitative research.

**Methods:**

Participants were selected using purposive sampling followed by theoretical sampling. Face‐to‐face semi‐structured interviews were conducted with fifteen patients diagnosed with chronic heart failure and three of their family members. Data analysis involved a three‐level coding technique of constant comparison to identify core categories.

**Results:**

The core category *Gaining Control of Life* was identified from the data, encompassing two main themes: factors influencing disease self‐management and self‐management strategies. Factors affecting disease self‐management were identified at three levels: individual, family and societal. Self‐management strategies applied by patients primarily focused on three domains: disease management, lifestyle adjustments and emotional regulation. Filial piety and familial care, combined with adequate and accessible local healthcare services, enhanced patients' sense of control, integrating disease management into their everyday lives.

**Conclusion:**

The self‐management experiences of patients with chronic heart failure represent an ongoing process of learning and adaptation, deeply influenced by socio‐cultural factors. Through this adjustment process, patients continuously develop their coping skills, ultimately achieving the goal of *gaining control of life*. Comprehensive and accessible healthcare and community support play a pivotal role in facilitating this adaptation. The findings of this study contribute to the enrichment of existing self‐care theories by highlighting the interplay between individual, familial and societal factors.

**Impact:**

This study aligns with and integrates key elements of Roy's Adaptation Model and Orem's Self‐Care Theory while emphasising the collaborative roles of patients, families and society in disease management.

**Implications for Patient Care:**

Enhance the quality of life of patients with chronic diseases, enabling them to take control of their lives more effectively.

**Patient or Public Contribution:**

Fifteen patients diagnosed with chronic heart failure and three family members agreed to participate in the study and shared their experiences with us.

## 1 Introduction

Chronic heart failure (hereinafter referred to as heart failure) affects 1%–2% of adults in high‐income countries (Martin et al. 2024). The condition predominantly occurs in older populations, with an average patient age of 64 ± 14 years (National Center for Cardiovascular Diseases 2022). Research indicates that approximately 40% of heart failure hospitalisations could be avoided through effective self‐management (He 2023).

Numerous studies have examined self‐management in chronic disease and identified it as a complex, multi‐factorial process. Key factors influencing patients' heart failure self‐care include their understanding of the disease, the presence of comorbid conditions and the availability of support from family, peers and the healthcare system (Metra et al. 2023; Kodama et al. 2020). Socio‐demographic elements such as economic status and health literacy can also affect patients' ability to manage their condition (Konlan and Shin 2023). Despite education and discharge planning, self‐management often remains suboptimal after hospital discharge (Jaarsma et al. 2021). Experts advocate for comprehensive interventions integrating personal, social and cultural perspectives to address these challenges and improve patient outcomes (Konlan and Shin 2023). This holistic approach acknowledges that sustainable self‐care practices must resonate with patients' daily lives and their community context.

Prior research on chronic heart disease management has highlighted several general trends. Many patients with heart failure experience moderate to low quality of life (Mulugeta et al. 2024). Some qualitative studies report that patients can successfully implement lifestyle changes, such as dietary modifications or exercise routines, as part of their self‐management (Gow et al. 2024; Schumacher et al. 2018). Other studies, however, describe patient resistance or difficulty maintaining these changes over time (Gow et al. 2024; Schumacher et al. 2018). Social determinants of health, including economic security, access to care and family support, play a significant role in shaping outcomes for heart failure patients (Powell‐Wiley et al. 2022). Most existing studies have been conducted in Western countries or lower‐resource settings; the extent to which findings generalise to affluent economies with strong healthcare infrastructure remains underexplored. Macau—a high‐income region with a unique blend of Chinese and Western cultural influences—provides an important but under‐studied context for understanding self‐management.

This study fills an important gap by using a grounded theory design to explore how patients with chronic heart failure in Macau manage their disease. The grounded theory research approach is ideal for uncovering processes and constructing an explanatory framework rooted in participants' lived experiences. In Macau's affluent, culturally unique setting, we seek to pinpoint the contextual factors, challenges and coping strategies that shape self‐management. Accordingly, our study will ask these questions: (1) How do patients in Macau with chronic heart failure experience daily life with their disease? (2) What difficulties do they encounter in managing their condition? (3) Which coping strategies do they employ to overcome these difficulties? Answering these questions will deepen our understanding of heart failure self‐care in a high‐resource Chinese context and inform the design of culturally tailored interventions for similar settings.

## 2 Methods

### 2.1 The Study Setting

Macau—a small, ultra‐dense, high‐income city where 92% of residents are ethnic Chinese—blends Eastern and Western cultures after centuries of Portuguese rule (Statistics and Census Service Office of Macau [SSM] 2022). Circulatory diseases cause 29.3% of all local deaths; cardiology admissions rose 18% between 2017 and 2022, and hospitals recorded nearly 1000 acute heart‐failure cases in the recent 3 years, with more than half in patients > 75 years (SSM 2023; LU et al. 2020). Given Macau's population size, these data imply an adult chronic heart failure prevalence of roughly 1%–2%, comparable to other high‐income regions. The indicators highlight the impetus of understanding how Macau residents live with heart failure and designing culturally tailored supports to strengthen their self‐management.

### 2.2 Study Designs

Grounded theory research is a well‐established approach and particularly effective for studying process‐oriented phenomena (Corbin and Strauss 2014). The self‐management practices of patients with heart failure evolve as part of their ongoing adjustment to living with the disease, making grounded theory an appropriate methodology for analysing this adaptation process. The study was followed by the Guideline for Reporting and Evaluating Grounded Theory Research Studies (Berthelsen et al. 2018) to improve the research quality and credibility.

### 2.3 Sampling and Recruitment

In the early stages of this study, purposive sampling was employed to recruit patients who could provide rich insights into their experiences of living with heart failure.

Eligible patients were ≥ 18 years old, had at least 1 year of heart‐failure treatment, and were New York Heart Association (NYHA) Class II–IV. NYHA divides patients into four classes (I–IV) based on how much physical activity they can perform before experiencing symptoms like shortness of breath, fatigue or palpitations: I = no limitation; II = slight; III = marked; IV = symptoms at rest. The 1‐year threshold and exclusion of Class I ensured participants had sufficient experience with moderate‐to‐severe disease to reflect meaningfully on self‐care. Patients with significant cognitive or hearing impairments were excluded because they may not provide reliable data. Participants were recruited at a major Macau hospital, where the primary researcher (the first author), Lam, a cardiovascular ward nurse, invited eligible in‐patients and outpatients to join the study during their admissions or clinic visits.

Theoretical sampling was adopted in the later stages of the study following the initial data analysis (Corbin and Strauss 2014). For example, the preliminary analysis revealed that patients' knowledge of heart disease significantly influenced their self‐management. Younger patients with higher education levels were specifically recruited in subsequent phases to further explore the relationship between disease‐related knowledge and self‐management behaviour.

To enrich the data, some patients' family members were also invited to participate in the study. The inclusion criteria for family members were: (Anfara Jr et al. 2002) living with the patient for at least 1 year and (Berthelsen et al. 2018) serving as the patient's primary caregiver. Family caregivers were approached by the primary researcher during their visits to the hospital and were invited to participate if they expressed interest in the study.

### 2.4 Data Collection

Semi‐structured interviews were conducted with both patients and their family caregivers. An interview guide was developed through a review of relevant literature. To refine the guide, a pilot interview was conducted with two patients and one caregiver, after which minor revisions were made. The finalised guide (Table 1) was used for all the interviews. Probing techniques were employed during the interviews to facilitate communication and gather in‐depth information. Interviews with caregivers were conducted after the patient interviews had been completed. These caregiver interviews were unstructured and focused on their perspectives on heart failure and their daily interactions with the patients, aiming to verify and supplement the information gathered from the patients. The rationale for using an unstructured format with caregivers was that their viewpoints and stories might not align neatly with the patient‐focused interview questions; giving them freedom to speak ensured we captured unanticipated insights about family dynamics and care.

**TABLE 1:** Interview guide.

How did you find out that you had heart failure? What were your feelings and coping measures when first experiencing symptoms of heart failure? What changes has the heart failure brought to your life? Such as changes in diets, exercise, emotions, as well as interactions with families and friends, etc. During your illnesses, how did you seek medical services? What are your plans and intentions in the future? What experiences do you have to share with other patients suffering heart failure?

Data collection took place from November 2021 to March 2022. The time and location of each interview were determined through mutual agreement between the primary researcher and the participants. Interviews were conducted in quiet environments, such as hospital wards or outpatient clinics. With participants' consent, the interviews were audio‐recorded using the Notability software. This software allowed the interviewer to take notes simultaneously during the recording, with the option to revisit and review the notes afterward. Immediately after each interview, the primary researcher recorded field notes, documenting the interview environment and his reflections on interacting with the interviewees.

After the twelfth interview no new properties, relationships or variations emerged, indicating theoretical saturation (Corbin and Strauss 2014). We then interviewed three more patients to verify that no additional insights appeared and, once confirmed, concluded data collection. The duration of patient interviews ranged from 36 to 79 min, with an average length of 54 min. Caregiver interviews ranged from 14 to 79 min, with an average length of 38 min.

### 2.5 Data Analysis

We began the data analysis by transcribing the interview recordings into Word documents. Each transcript was then meticulously reviewed line by line, and content reflecting patients' experiences with heart failure was identified and coded through a process known as open coding (Corbin and Strauss 2014). Next, we grouped similar open codes into categories based on conceptually related meanings during axial coding. As the axial codes were continuously integrated and refined, they became increasingly condensed, culminating in the identification of a core category that represented the self‐management experiences of patients with heart failure. The data analysis process was facilitated using NVivo 11 Plus software.

### 2.6 Ethics Considerations

The study was approved by both a university's Research Management and Development Department (REC‐2021.16) and a hospital ethics committee (2021‐030) and complied with the Declaration of Helsinki for research studies involving humans (World Medical Association 2013). Prospective participants received a detailed verbal and written briefing on aims, procedures of the study and risks/benefits of study participation. The written informed consent was obtained before the interviews were conducted. During the interviews, the participants could refuse questions, pause or withdraw without adverse results to the care or services they would receive. The primary researcher monitored the signs of distress during the interviewing and reminded participants they could skip uncomfortable questions. A staff psychologist was available for referral if needed, although no referrals were required. To safeguard privacy, all identifiers were removed; pseudonyms were assigned, and audio files and transcripts were stored on an encrypted, password‐protected computer that was accessible only to the researchers; hard‐copy materials were kept in a locked cabinet.

### 2.7 Rigour

This research project was part of a thesis for the Master of Nursing (MN) programme. To align with Anfara, Brown and Mangione's four benchmarks for qualitative rigour—credibility, dependability, confirmability and transferability (Anfara Jr et al. 2002), we embedded a layered quality‐assurance strategy under continuous oversight by the MN programme committee. Credibility was pursued through maximum‐variation sampling (patients spanning six decades of age, both sexes, varied educational and income levels, plus three co‐resident caregivers) to ensure the emergent theory reflected a broad spectrum of experience. We then used two credibility checks: (a) member checking, in which five randomly chosen participants reviewed full transcripts and affirmed our interpretations; and (b) investigator triangulation, whereby the primary researcher (Lam, the MN student) and his supervisor (Mao, the second author) independently coded early transcripts, compared codebooks line‐by‐line and resolved discrepancies through analytic dialogue, refining definitions until intercoder agreement exceeded 90%. Dependability was addressed with a meticulous audit trail: encrypted audio files, verbatim transcripts, successive codebook versions, dated analytic memos and regular meetings between the researchers. Six weeks after initial coding, the primary researcher recoded 20% of transcripts and congruent coding patterns confirmed stability of interpretations over time. Confirmability was strengthened through ongoing reflexive journaling and formal bracketing sessions held before data collection and midway through analysis; the primary researcher explicitly documented personal assumptions (e.g., a clinical bias toward viewing family support as uniformly beneficial) and actively sought disconfirming evidence, thereby anchoring findings in participants' voices rather than researchers' preconceptions. Finally, transferability was promoted by providing thick contextual detail—setting, healthcare system, cultural norms—and extensive verbatim quotations, enabling readers to judge the applicability of our grounded theory approach to other high‐income, Chinese‐heritage contexts.

## 3 Findings

### 3.1 Participants

Among the 15 patients with chronic heart failure, seven were male and eight were female, with ages ranging from 29 to 91 years. The duration of their heart failure varied from 1 to 40 years. Over half of the patients were NYHA Class II, and eight had a left ventricular ejection fraction > 45%. Thirteen patients were married, all of whom had children. Most patients lived with their family members and could care for themselves independently. Two of the three caregiver participants were male, and the three were aged 43–93 years. They had lived with the patients for over 30 years.

Through constant comparison of the interview data, two major categories emerged: factors influencing self‐management and self‐management strategies. These categories highlighted the multiple factors impacting patients' self‐care within the community and the coping measures they employed to address these challenges.

### 3.2 Factors Influencing Self‐Management

#### 3.2.1 Personal Factors

Personal factors primarily involve patients' perceptions of their disease, concerns about its impact on their families and personal life expectations. Patients acquired disease‐related knowledge through various channels, prioritising treatment information, followed by details on medications, diets and coping strategies for daily life. Family Member 2 shared: ‘He (the patient) is very nervous about his disease and looks for information online by himself. He has prepared everything’.

Patients often expressed a desire to minimise the impact of their condition on their daily lives, striving for independence without relying on family support. Patient 7 explained their proactive approach to seeking treatment: ‘It is really a pity when you need someone to take care of you. So, I think treatment should be sought earlier’. Some patients actively sought treatment to fulfil personal goals, such as witnessing significant milestones in their children's lives. Patient 9 stated: ‘Now I am having regular follow‐up visits and check‐ups… I want to see my son graduate from university, get married and have children’.

#### 3.2.2 Family Factors

Among the 15 patients, 14 lived with their families. Family members played an integral role in supporting patients' daily self‐management routines. As heart failure progressed, the patients' daily activities were restructured, with responsibilities shared between the patients and their families. Patients undertook tasks they could manage, while family members assisted with more challenging ones. Patient 10, who had limited activity tolerance, described the support provided by his family: ‘I don't have to do housework at home now. My son helps me; he cooks and washes the dishes for me’.

Family members often understood the specific types of assistance patients required and adjusted their roles accordingly. However, patients also retained control over decisions about the help they needed, particularly from their children. For instance, Patient 2 shared: ‘My daughter takes me to see the doctor, and my son‐in‐law drives me to the health center to get my insulin injections…I have a bell, and when something happens, I ring to call my children over’. This highlights the patients' sense of control over their disease management and the authority they maintained in their relationships, especially as elderly members of their families.

#### 3.2.3 Social Factors

Patients generally reported that accessing medical care in Macau was convenient, with readily available services. Residents over 65 were eligible for free medical care, alleviating concerns about medical expenses. Regular visits to medical institutions and interactions with healthcare professionals also provided opportunities for the patients to obtain information and adjust treatment plans on time. Patient 7 mentioned: ‘I have quite a few things to ask the doctor, who says he doesn't mind meeting me. I can talk to him a bit longer. So, I regularly go to see him, looking for solutions to the difficulties I encountered in life’.

Social and medical home services were invaluable for patients unable to care for themselves fully. For example, Patient 1, who had suffered from heart failure for 9 years and lived alone, was assigned to a government‐provided elderly apartment, sharing her story: ‘I am assigned to an elderly apartment, where someone delivers meals to me. A physical therapist often comes to my apartment for physical therapy. Someone from a social welfare agency also visits me, providing me with daily necessities like clothes hangers and canes’.

### 3.3 Self‐Management Strategies

Patients recognised that, despite the support of their families, medical professionals and social institutions, the responsibility for managing their disease ultimately rested with them. Over the course of their conditions, patients gradually developed an understanding of their disease characteristics and implemented self‐management strategies they believed appropriate. These strategies focused on disease management, lifestyle adjustments and emotional regulation.

#### 3.3.1 Disease Management

Medication adherence was an integral part of patients' routines. Most patients were highly aware of their medication regimens and demonstrated good adherence, often employing personalised methods to ensure doses were not missed. Patient 8 shared: ‘I usually split medications into several portions using small bags. I won't forget to take my medicines because I write them here. If I miss a dose, I can find it out’.

Patients also developed a certain understanding of their disease's characteristics. When changes occurred in their condition that did not significantly impact their physical functions, they adjusted their medications based on past experiences and advice from healthcare providers. Patient 14 explained: ‘The doctor said that if my heart rate was high, I could take an extra arrhythmia pill. I measure my blood pressure daily, and when I see my heart rate over 100, I take an additional pill’.

Family members also participated in monitoring the patients' conditions. Over time, they became familiar with the patients' health status and offered supportive advice. Family Member 2 noted: ‘In 2019, his cardiac ejection fraction was only 21%. Recently, during stable periods, it has reached 37% to 38% after we did cardiac ultrasounds’.

#### 3.3.2 Lifestyle Adjustments

Most patients made significant lifestyle changes as their disease progressed, particularly in their diets. Adjustments included adopting low‐sodium diets, restricting fluid intake and establishing new meal habits. Since these patients lived with family members, such lifestyle changes were often supported by their families, who cooperated in making adjustments and, in some cases, made sacrifices. Patient 9 mentioned: ‘My family knows that I need a low‐sodium diet because of my disease. We eat together, and sometimes they eat lighter meals. If they want to eat something I can't have, they will eat separately, so we eat differently’.

Almost all patients were willing to follow doctors' advice and make behavioural changes, such as quitting smoking or adopting exercise routines, to manage their condition better. Patient 13 shared: ‘I didn't exercise before getting this disease, but now I insist on riding a bike every day, setting a calorie‐burning goal’.

#### 3.3.3 Emotional Regualtion

Patients generally acknowledged that maintaining a positive attitude was beneficial for controlling disease progression. However, they admitted experiencing emotional instability as their disease advanced. When negative emotions arose, patients became aware of their mental state and actively sought ways to adjust their mindset. Patient 7 reflected: ‘I might be a bit worried about the heart disease and its ultimate development. I tell myself not to think too much about the prognosis. You have to trust what the doctor says and stop overthinking’.

Some patients coped by talking to those around them, listening to music or seeking psychological counselling. Patient 4 stated: ‘The psychologist made me more positive…Perhaps the medications made me more willing to speak to others. My husband has only just begun to understand my disease’.

### 3.4 Core Category

As discussed earlier, patients continuously adapted and learned to coexist with their diseases, leveraging support from their families and society to create an environment conducive to disease control while maintaining their social functioning. For patients, their disease did not define their entire life. Instead, they sought to minimise its impact, actively making changes to achieve control over their condition and ultimately reclaim dominance over their lives.

**FIGURE 1:**
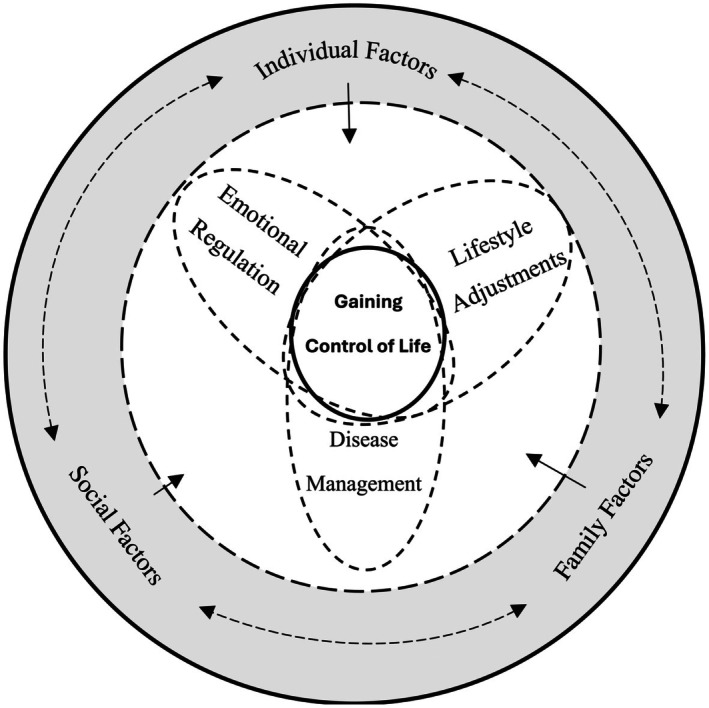
The influencing factors and coping strategies related to the self‐management of patients with chronic heart failure.

Thus, ‘gaining control of life’ was identified as the core and essence of a patient's self‐management. It illustrates that disease management is a socialised process shaped by interactions between patients and their environment. Figure 1 illustrates the theoretical framework that outlines the influencing factors and coping strategies related to the self‐management of patients with chronic heart failure.

## 4 Discussion

Although there is a relatively rich body of literature on the self‐management of patients with chronic diseases, this study employed a qualitative research methodology to deeply explore the experiences of patients with chronic heart failure within the specific social context of Macau. It enriches understanding of commonalities and differences in self‐care among chronic patients across diverse cultural settings.

Coping with disease is a learning process for patients, allowing them to rediscover themselves and learn to coexist with their disease. To maintain or improve their quality of life, patients strive to manage their physical and mental health through various strategies, including disease management, lifestyle adjustments and emotional regulation. These coping strategies extend beyond the disease management to include comprehensive lifestyle changes. Such changes often involve adjustments in the entire family's lifestyle (White‐Williams et al. 2020).

During chronic disease progression, family functioning often strengthens, with family members becoming vital resources for managing the disease and serving as a source of internal motivation for the patient (Herber et al. 2021). This is particularly relevant within Chinese cultural contexts, where identity is strongly tied to family relationships, and individuals often see themselves as part of a collective unit (Wong 2023). While disease and seniority may grant patients the cultural rights to demand care from younger family members (Fei 1992), the participants in this study generally expressed a desire not to burden their families. Instead, they wished to witness significant life milestones of their children, such as graduation and marriage, which they viewed as fulfilling their parental responsibilities. It can be seen that Macau remains deeply rooted in traditional Chinese culture despite its historical Western influences. The presence of disease in this context often stimulates and highlights filial piety and care within conventional family dynamics.

Conventional guidelines for chronic disease management typically emphasise individual‐level factors, such as regular physical activity, adherence to healthy diets, smoking cessation and weight management (Sigamani and Gupta 2022). However, this study highlights the important role of comprehensive and accessible community support in maintaining or even enhancing patients' self‐care capacities. Participants in this study frequently interacted with the healthcare system, regularly visited medical institutions for diagnoses and treatments and utilised home medical and social services. These experiences reflect the richness of Macau's healthcare resources.

Such comprehensive medical and social support provides patients with a sense of security and helps enhance their knowledge of care and their ability to manage at home (Herber et al. 2021). These geographical and social welfare advantages further support patients in managing their conditions. National data corroborate this, showing reduced mortality among middle‐aged and older patients with sufficient social support (Powell‐Wiley et al. 2022). The findings of this study underscore the importance of local community support combined with national policies that enhance healthcare access and resources.

The experiences documented here are grounded in Macau's particular socio‐cultural milieu. Patients in other high‐income Asian regions (like Hong Kong or Singapore) might exhibit similar patterns of strong family involvement and good healthcare support, whereas those in low‐income or rural areas would face different constraints. Nevertheless, some insights are likely broadly relevant: the central importance of patients striving for normalcy and control, the value of family support in chronic disease management and the need to address emotional as well as physical aspects of self‐care are themes echoed in the wider literature (Dalhammar et al. 2023; Kleman et al. 2024). Healthcare professionals and researchers in other contexts can compare their local conditions to Macau's as described to gauge how applicable these results might be. For instance, in societies where family ties are weaker or healthcare access is poorer, patients might rely more on peer support or personal resilience, and interventions might need to focus more on building external networks of support.

This study has contributed to and supplemented existing nursing theories, particularly Roy's Adaptation Model (RAM) and Orem's Self‐Care Theory (OSCT). RAM conceptualises humans as dynamic adaptive systems that respond to external stimuli through physiological and cognitive regulation. It emphasises holistic patient assessments across four major stimulus factors: physiological function, self‐concept, role function and interdependence, with interventions tailored based on practical or ineffective responses (Roy 1999). OSCT focuses on concepts such as self‐care, self‐care deficits and nursing systems, asserting that patients should maintain self‐care capabilities. When these capabilities decline to the point where daily needs cannot be met, appropriate care assistance becomes necessary (Orem 1991). Our findings confirm that the self‐management of patients with heart failure is a patient‐centred process involving adapting the patient's entire environment. It also reveals the strong desire of patients with chronic diseases to maintain self‐care capabilities by maximising resources from families and the social environment. In short, this study aligns with and integrates key elements of both RAM and OSCT while emphasising the collaborative roles of patients, families and society in disease management. In the self‐management of heart failure, the importance of family and social support cannot be overstated, offering new perspectives for understanding and applying existing self‐management theories.

### 4.1 Strengths and Limitations

This study's strengths lie in its rigorous qualitative design and contextual depth. By using a grounded theory approach, we were able to develop a theoretical framework directly grounded in participants' experiences, providing a holistic view of the self‐management process. The qualitative methodology allowed for rich data collection, capturing not just what patients do to manage heart failure, but *how* and *why* they do so within their life context.

Several factors limit the study's generalisability. The 15‐patient sample, drawn from one hospital and dominated by NYHA Class II–III cases (only one Class IV), under‐represents end‐stage heart failure and may miss challenges unique to severe disease; larger, more heterogeneous samples are required in future research. Moreover, the cross‐sectional, single‐interview design relies on recall, obscuring how self‐management changes over time—longitudinal follow‐up would capture that evolution more accurately (Tuthill et al. 2020).

## 5 Conclusion

Chronic heart failure is a long‐term process that profoundly affects patients' quality of life. This study reveals the richness and diversity of patient experiences in different social contexts. It is found that during coexistence with diseases, patients actively learn how to live with their conditions. Chinese family culture plays a key role in patients' self‐management, where patients and their family members often prioritise each other's needs, making family factors an important pillar in their coping with diseases. Furthermore, convenient and affordable social resources further enhance patients' self‐management abilities. As a result, self‐management is a patient‐centred team operation model, where family and social resources facilitate patients' self‐management capabilities. Future healthcare policies should focus on family‐centred strategies and further improve community support systems, which will help enhance the quality of life of patients with chronic diseases, enabling them to take control of their lives more effectively and achieve the best quality of life.

## Author Contributions

**Kakit Lam:** conceptualisation, data curation, formal analysis, investigation, methodology, software, validation, visualisation, writing – original draft preparation. **Aimei Mao:** conceptualisation, data curation, methodology, supervision, validation, writing – review and editing.

## Conflicts of Interest

The authors declare no conflicts of interest.

## Data Availability

The data that support the findings of this study are available on request from the corresponding author. The data are not publicly available due to privacy or ethical restrictions.

## Appendix 1

### Interview‐Related Diary

#### General Information

- Patient: Female, 78 years old, no formal education, retired.
- Medical Classification: NYHA Functional Class II.
- Heart Failure History: 9 years.
- Living Situation: Lives alone, partially self‐sufficient with assistance from two caregivers (both female).

#### Medical History

- Heart failure, atrial septal defect, moderate mitral regurgitation (MR), vertebrobasilar insufficiency, hypertension, diabetes mellitus, post‐PCI, secondary pulmonary hypertension, right humeral fracture, total thyroidectomy, right ureteral carcinoma surgery, liver cyst.

#### Disease History

- 2010.6.21–2010.7.1: Hospitalised for chest tightness and dizziness.
- 2012: Experienced shortness of breath during activity. ECHO results: moderate tricuspid regurgitation (TR), EF 68%. Consulted a private doctor.
- 2019.2.25–2019.3.9: Hospitalised for shortness of breath during activity. BNP > 35,000; ECHO: left atrium enlargement, impaired left ventricular diastolic function, EF 54%. CXR: pulmonary congestion.
- 2019.10.17–2019.11.01: Hospitalised for chest tightness and shortness of breath. ECHO: secondary atrial septal defect, moderate mitral regurgitation (MR).
- 2019.11.23–2019.11.26: Hospitalised for chest tightness and shortness of breath.
- 2020.3.13–2020.3.24: Hospitalised for chest tightness and shortness of breath.
- 2021.5.28–2021.6.27: Hospitalised. ECHO: left ventricular enlargement, EF 48%. CXR: pulmonary congestion, bilateral lower lung infiltrates.
- 2021.11.14–2021.12.3: Hospitalised for chest tightness and shortness of breath. BNP 7046; ECHO: EF 30, left atrium enlargement, atrial septal defect.

#### Interview Details

- Date of Interview: November 19, 2021.
- Duration: 1 h 18 min.
- Participants:
- 1 respondent (patient) in a hospital uniform.
- 1 researcher in a nurse's uniform.
- 1 instructor (the supervisor of the researcher) in a work uniform.
- Setting: in a private patient room with a quiet environment and clear acoustics. Participants sat opposite each other in a relaxed, conversational setup conducive to informal discussion.

#### Summary of Key Insights

The respondent was talkative and quickly engaged in the interview from the start. However, as the interview progressed, the respondent's focus and energy began to wane, possibly due to the extended duration. Despite this, the overall mood and engagement throughout the interview was positive.

1. Disease Awareness and Cognition
  - Information Sources: The patient showed a willingness to receive disease‐related information, mainly relying on doctors, followed by people around them and personal experience. However, the patient expressed doubts about the accuracy of online health information.
  - Knowledge on disease: Despite the willingness to learn, the patient demonstrated limited knowledge of heart failure symptoms, daily treatments, dietary requirements and medications, relying more on empirical judgement.
  - Adherence to Treatment: Fear of burdening family members motivated the patient to maintain high adherence to medical treatments. She actively sought medical care and monitored her condition when symptoms arose.
2. Self‐Management Strategies
  - Daily life Adjustments: The patient implemented lifestyle changes, including adhering to a medication schedule, increasing medical visits and developing habits to cope with heart failure. Initially, her condition affected sleep, but she has since learned to regulate her emotions to manage stress more effectively.
  - Emotional Regulation: The patient avoided negative emotions, actively driving herself to maintain a positive outlook. She described feeling emotionally stable, with minimal fluctuations in mood.
3. Support Systems
  - Family Support: The patient noticed changes, including assistance with meal preparation, household chores, medication management and medical appointments. According to the patient, her family members also provided financial and material support, such as assistive devices.
  - Social Welfare Support: The patient received assistance from social welfare organisations, including follow‐up consultations, emergency support system installations and material provisions. However, the patient noted limitations, such as insufficient manpower for frequent accompaniment to medical appointments and a lack of emotional support services.
  - Community and Government Support: The patient accessed government‐subsidised housing, financial aid for daily living, long‐ and short‐term medication subsidies and hospitalisation support.
  - Peer Support: Friends provided emotional and financial support, as well as emergency assistance, such as safety bell installations. Friends also frequently checked on the patient's condition, both in person and online.

### Conclusion

This interview highlighted the patient's proactive approach to self‐management, driven by a fear of burdening others and supported by family, community and government resources. However, gaps in disease knowledge and limited access to emotional support services remain barriers to achieving optimal disease control.

## Appendix 2

### An Example of Data Analysis

**Table:**

Question 7: That's how people thought about you when you were well. But how do you think they have perceived you after you've been ill for some time?	
Respondent (seems nervous, avoiding eye contact with the researcher): Because now I'm seeing a mental health center, and the psychologist told me things like, ‘Do what you enjoy doing, and meet the people you like to meet’. At the school, some colleagues have been helping me. They understand me. Some colleagues said, ‘I understand how you feel. If there's anything you can't do, let us know, and we'll help you’. But there are also some colleagues who don't understand me. So, I don't care much about what they said. One colleague, who is a beliver from a church, told me to forgive those who said unappropraite things to me. Well, I am not feeling strongly for the way people treat me.	Finding Your Own Support: Emotions: Seeking counselling from a psychologist, transitioning from initially keeping things private to now sharing with familiar colleagues. Warmth from Friends: Support: Colleagues offering comfort.
Question 8: How did organisations or the school help you?	
Respondent (choking up, tears in the eyes): After I spoke about my conditions to the heads in the school, they suggested reducing some of my teaching task. I feel that the school has really been there for me, and I'm very grateful. Researcher: So, they've been supportive of you. Respondent (choking up, tears in the eyes): Yes, the school has helped me. The principal and directors are very supportive. They even held a special meeting to discuss on how to help me.	Support from Community Organisations: Work Environment: Reduction of the workload.
Question 9: You mentioned that you have visited a mental health center. When did you first start feeling stressed?	
Respondent: I think it started when my condition worsened. On top of that, the pandemic made me very unhappy because I couldn't cross the border. I couldn't go to Zhuhai (a city in mainald bordering with Macau). In previous times, my husband and I would go to our home in Zhuhai every Sunday for the weekend.	Long COVID—long‐term adverse impacts:
Also, during the pandemic, I didn't dare get the vaccination because of my heart condition.	Restricted Travel: Leading to the loss of previous relaxation measures.
Researcher: I understand, arrhythmia.	Health Condition
Respondent: My condition is unstable and the heart beats sometimes fast and sometimes slow. The hospital on the hill (referring to the public hospital) didn't dare give me the vaccine. So, while others needed a nucleic acid test once a week, I had to have twice. Because I had to go to the hospital and also do the test. This has caused me a lot of anxiety.	Heightened anxiety during the pandemic.
